# Point-of-care Ultrasound Clarified the Diagnosis of an Occipital Artery Pseudoaneurysm After Blunt Trauma

**DOI:** 10.5811/cpcem.38448

**Published:** 2025-05-10

**Authors:** Kahra Nix, Sydney Johnson, Daniel Perling, Brandon Parkinson, Haely Studebaker, Brett Foster

**Affiliations:** University of Louisville School of Medicine, Department of Emergency Medicine, Louisville, Kentucky

**Keywords:** blunt head trauma, point-of-care ultrasound, pulsatile mass, pseudoaneurysm, occipital artery

## Abstract

**Case Presentation:**

A 54-year-old male presented to the emergency department one month after blunt trauma to the head complaining of two weeks of worsening swelling over his right posterior scalp. Computed tomography of the head without contrast showed a soft tissue lesion. Point-of-care ultrasound (POCUS) was performed to clarify the soft tissue lesion that was found on computed tomography and revealed an occipital artery pseudoaneurysm.

**Discussion:**

An occipital artery pseudoaneurysm is a rare diagnosis. A POCUS performed by the emergency physician ensured an accurate and timely diagnosis for this patient.

## CASE PRESENTATION

**Image 1:**
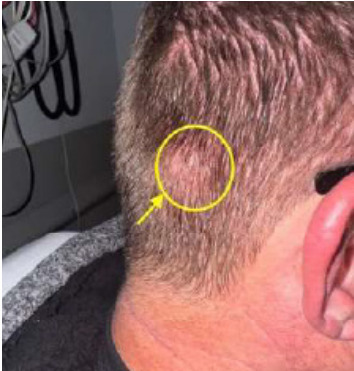
A patient reported an expanding swelling over his scalp with a physical exam revealing a non-tender, two-centimeter pulsatile mass (yellow circle with arrowhead) with overlying erythema on the right occipital scalp.

**Image 2:**
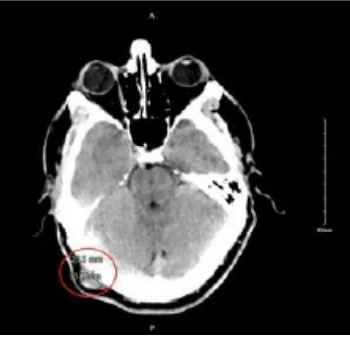
Axial computed tomography of the head without contrast demonstrating a focal, soft-tissue lesion abutting the intact calvarium, measuring 2.0 x 1.3 centimeters that was isodense to the surrounding muscle (red oval).

**Image 3A:**
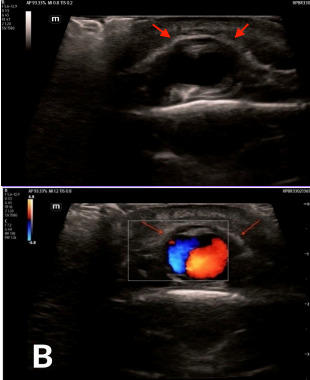
Point-of-care ultrasound (POCUS) gray-scale image using a linear probe demonstrates an anechoic, cystic structure (red arrowheads) adjacent to the occipital artery representing a pseudoaneurysm. **Image 3B**. POCUS image using a linear probe with color Doppler demonstrates turbulent flow with the characteristic yin-yang appearance within an anechoic, cystic structure (red arrowheads) adjacent to the occipital artery confirming the presence of a pseudoaneurysm.

A 54-year-old male with a past medical history of hypertension and depression presented to the emergency department (ED) one month after a physical assault with the complaint of swelling over his posterior scalp without neurological deficit. He had initially noticed it two weeks prior, but it was expanding. Physical exam revealed a non-tender, two-centimeter (cm) pulsatile mass with overlying erythema on the right occipital scalp (Image 1). Computed tomography (CT) of his head without contrast was performed and was negative for skull fracture or any intracranial pathology, but the study showed a focal, soft tissue lesion abutting the intact calvarium measuring 2.0 x 1.3 cm (Image 2). Point-of-care ultrasound (POCUS) was performed by the emergency physician to clarify the soft tissue lesion found on CT. Gray-scale images showed an anechoic, cystic structure (Image 3a) that was pulsatile with turbulent flow seen with the characteristic yin-yang appearance on color flow Doppler examination (Image 3b).

## DISCUSSION

Images 3a and 3b describe the classic ultrasound findings of a pseudoaneurysm. These POCUS findings further prevented consideration of bedside incision and drainage in the ED of this erythematous, soft-tissue swelling, which carried risk of mortality and morbidity for this patient. Neurosurgery admitted the patient for further management after reviewing the POCUS and CT images. Digital subtraction angiography was performed to clarify the right occipital artery pseudoaneurysm and to determine the appropriate management. Then neurosurgery performed transcatheter glue embolization with N-butyl cyanoacrylate for definitive management.

*CPC-EM Capsule*

What do we already know about this clinical entity?

*An occipital scalp pseudoaneurysm is a rare diagnosis that can be made by duplex ultrasound*.

What is the major impact of the image(s)?

*These easily obtained ultrasound images detail the classic appearance of a pseudoaneurysm with the clarity of color Doppler to display turbulent flow with a yin-yang pattern*.

How might this improve emergency medicine practice?

*Point-of-care ultrasound performed by a physician to evaluate a pulsatile mass has the potential to expedite care for and prevent missing a diagnosis like a pseudoaneurysm*.

An occipital artery pseudoaneurysm is a rare diagnosis likely due to protection for the artery from trauma by surrounding scalp musculature, and it often has a delayed presentation.^1^,^2^ However, there are other causes for this diagnosis, beyond trauma, such as head and neck procedures.^1^,^2^ An injury to the arterial wall leads to a hematoma formation and eventually turbulent blood flow between the artery and the adjacent, communicating pseudoaneurysm.^1^–^3^ During the initial workup, duplex ultrasound, angiography, or both are useful in ensuring the prompt diagnosis of a pseudoaneurysm.^3^,^4^
